# Speech Production Intelligibility Is Associated with Speech Recognition in Adult Cochlear Implant Users

**DOI:** 10.3390/brainsci15101066

**Published:** 2025-09-30

**Authors:** Victoria A. Sevich, Davia J. Williams, Aaron C. Moberly, Terrin N. Tamati

**Affiliations:** 1 Department of Speech and Hearing Science, The Ohio State University, Columbus, OH 43210, USA; tamati.1@osu.edu; 2 Department of Otolaryngology, Vanderbilt University Medical Center, Nashville, TN 37232, USA; davia.j.williams@vanderbilt.edu (D.J.W.); aaron.c.moberly@vumc.org (A.C.M.)

**Keywords:** speech production, speech perception, cochlear implants, phonological processing

## Abstract

**Background/Objectives**: Adult cochlear implant (CI) users exhibit broad variability in speech perception and production outcomes. Cochlear implantation improves the intelligibility (comprehensibility) of CI users’ speech, but the degraded auditory signal delivered by the CI may attenuate this benefit. Among other effects, degraded auditory feedback can lead to compression of the acoustic–phonetic vowel space, which makes vowel productions confusable, decreasing intelligibility. Sustained exposure to degraded auditory feedback may also weaken phonological representations. The current study examined the relationship between subjective ratings and acoustic measures of speech production, speech recognition accuracy, and phonological processing (cognitive processing of speech sounds) in adult CI users. **Methods**: Fifteen adult CI users read aloud a series of short words, which were analyzed in two ways. First, acoustic measures of vowel distinctiveness (i.e., vowel dispersion) were calculated. Second, thirty-seven normal-hearing (NH) participants listened to the words produced by the CI users and rated the subjective intelligibility of each word from 1 (least understandable) to 100 (most understandable). CI users also completed an auditory sentence recognition task and a nonauditory cognitive test of phonological processing. **Results**: CI users rated as having more understandable speech demonstrated more accurate sentence recognition than those rated as having less understandable speech, but intelligibility ratings were only marginally related to phonological processing. Further, vowel distinctiveness was marginally associated with sentence recognition but not related to phonological processing or subjective ratings of intelligibility. **Conclusions**: The results suggest that speech intelligibility ratings are related to speech recognition accuracy in adult CI users, and future investigation is needed to identify the extent to which this relationship is mediated by individual differences in phonological processing.

## 1. Introduction

Cochlear implants (CIs) provide access to spoken language for adults with moderate-to-profound hearing loss (HL), but speech communication outcomes are highly variable [1,2,3,4,5]. While some CI users achieve excellent speech recognition outcomes, others continue to report significant difficulties understanding speech, particularly in real-world, challenging listening conditions [4,6,7]. Standard clinical outcome assessments typically focus on speech recognition, often using isolated words or sentences presented in quiet or noise. While these measures provide valuable insight into auditory processing, they likely do not capture individual differences in broader communication ability. Successful communication via spoken language requires that individuals not only accurately perceive speech but also can accurately produce speech. However, speech production outcomes have received less attention than perception outcomes among adult CI users, and the extent to which speech production reflects individual differences in speech perception and processing remains unclear. Understanding the relationship between speech production and perception outcomes may offer new insights into the underlying mechanisms that shape communication outcomes in adult CI users. In this study, we examined whether individual differences in speech production, measured perceptually and acoustically, are associated with speech recognition scores in experienced adult CI users.

### 1.1. Auditory Feedback Influences Speech Production

Auditory feedback refers to the process by which an individual receives sensory feedback regarding their own speech. Auditory feedback plays a critical role in influencing speech production, both in real time and over a longer time scale. In the short term, auditory feedback mechanisms help talkers detect and correct speech errors in real time, facilitating the intelligibility of their speech. In the long term, broader auditory experiences and auditory input across the lifespan (which include auditory feedback) support the maintenance and refinement of phonological representations, or a listener’s mental representation of speech sounds. In individuals with normal hearing (NH), ongoing, long-term access to detailed auditory input supports the formation and refinement of robust phonological representations. Phonological representations serve as a foundation for both the perception and production of speech, allowing for accurate recognition of speech sounds and clear and intelligible articulation, e.g., [8,9,10].

Evidence for a link between speech perception and production is robust, e.g., [11,12]. For example, real-time experimental modifications to auditory feedback consistently drive talkers to make online modifications to their own speech in response to these modifications [13,14,15,16,17,18]. The study in [14] instructed talkers to produce a series of consonant–vowel–consonant (CVC) sequences and modified, in real time, the first and second formant frequencies (F1 and F2) of one of the vowels (/ɛ/) produced by the talkers. Over the course of the experimental session, this altered feedback led to modifications to their productions not only of /ɛ/ but also of the other vowels tested (/i, ɪ, æ, ɑ/). These findings support the notion that NH listeners make use of short-term auditory feedback to adjust productions of their own speech in real time. In addition, the articulatory adjustments were made not only to the vowel whose formants were manipulated but also to the other vowels in each talker’s vowel space. These modifications to all vowels in the vowel space indicate that talkers can modify vowel production to maintain phonetic contrast among vowels. While most of these experiments manipulated short-term auditory feedback, changes to auditory feedback over longer periods of time may also shape phonological representations if altered feedback is sustained.

For individuals with CIs, auditory input is spectrally degraded and lacks the fine-grained acoustic detail that supports detailed phonological representations. In post-lingually deafened adult CI users—i.e., CI users who acquired hearing loss after learning their first language—cochlear implantation often follows a long period of auditory deprivation in the form of sensorineural HL [19]. This reduced auditory fidelity of the signal delivered by the CI following a period of auditory deprivation can reduce the precision of phonological categories over time, in part by limiting the quantity and quality of auditory feedback [20,21,22,23,24,25,26]. Despite this degraded auditory feedback, however, many CI users use this feedback in real time to monitor their speech as it is produced. Studies that have examined changes in speech production between conditions where CI users have their processors on (i.e., auditory feedback was available) vs. when they take their processors off (i.e., no auditory feedback was available) have observed changes in acoustic speech parameters as a result of these short-term modifications to auditory feedback [27,28,29,30,31,32]. Collectively, evidence from these studies suggests that even shortly after CI activation many CI users take advantage of auditory feedback to shape their speech production in real time, potentially to facilitate the intelligibility of their own speech.

The results reviewed above are consistent with models that argue for the importance of auditory feedback for the development and maintenance of intelligible speech. For instance, the Directions Into Velocities of Articulators (DIVA) model of speech motor control [33,34,35] posits a direct link between auditory feedback and motor control of speech articulators. Specifically, the DIVA model proposes the existence of a neurocognitive representation of the expected auditory consequences of a spoken utterance in addition to an acceptable range of variation for that utterance. According to DIVA, if the incoming acoustic signal of a talker’s own utterance does not fall within this acceptable range of variation, a feedback control system initiates corrective motor signals that the somatosensory system translates into movements to correct speech errors or to otherwise enhance speech production (e.g., by hyperarticulating speech when asked to repeat it). Further, the DIVA model proposes that the targets of speech utterances are auditory, rather than articulatory. Therefore, under this framework, long-term limitations in auditory feedback could impact speech production through a combination of reduced motor control of articulators and imprecise auditory targets of speech gestures. Intact phonological representations are critical for maintaining auditory targets, underscoring the importance of phonological processing (i.e., the ability to access, compare, and manipulate speech sounds) for both the perception and production of spoken language.

### 1.2. Phonological Processing for Speech Perception and Production

A large body of research across diverse populations has provided evidence that more accurate speech perception is associated with more precise or intelligible speech production [36,37,38,39,40,41,42,43]. For example, Ref. [42] found that NH participants who were more accurate in discriminating between vowel contrasts in perception also produced a given pair of vowels with more phonetic contrast than those who were less accurate in discrimination. This relationship between perception and production may be especially relevant for CI users, whose perception and production systems must adapt to a degraded input signal. Phonological processing supports both processes. Phonological processing tends to be poorer in adults with HL relative to adults with NH, likely due to long-term reductions in and changes to auditory input during long periods of deafness [20,22,23,24,26,44,45,46,47]. In CI users specifically, weaker phonological processing skills may limit the ability to extract detail from the speech signal, resulting in difficulties recognizing phonemic differences across stimuli and in producing clearly differentiated speech sounds [22,24,25,26,48,49,50,51]. Collectively, these findings demonstrate that auditory deprivation leads to modifications in the sound structure of the lexicon among adults with HL with and without CIs and that these modifications have consequences for both speech production and perception.

Because modified phonological representations affect both the production and perception of spoken language, both production and perception measures can be used to assess the quality of phonological representations. One way of indirectly assessing phonological representations in CI users is through timed single-word reading tasks. These reading efficiency tasks provide an indirect measure of phonological representations by assessing processing efficiency, which reflects the quality of underlying representations. More specifically, reading efficiency relies on phonological decoding processes and direct access to the sound structure of the lexicon, both for nonwords and real words [52,53,54]. In a study assessing phonological processing in experienced adult CI users, Ref. [51] found that CI users with better phonological processing efficiency, reflected by higher word reading scores, demonstrated higher accuracy on a measure of sentence recognition than CI users with relatively poorer phonological processing. Substantial individual differences in phonological processing were observed among the CI users in their study, suggesting that even experienced adult CI users show variability in the efficiency through which they process and encode speech sounds. The implications of this variability have been explored to some extent using measures of speech perception in adult CI users [24,25,49,55], but the extent to which individual differences in speech production are influenced by phonological processing ability are not well understood.

### 1.3. Acoustic and Perceptual Measures of Speech Production in CI Users and Their Relation to Speech Perception

Despite growing interest in the cognitive factors that contribute to individual differences in CI speech recognition outcomes, the relationship between speech production and perception remains relatively underexplored in adult CI users. Most research has focused on speech recognition performance, with relatively little attention paid to how speech production quality relates to perceptual and cognitive–linguistic abilities. Some studies suggest that both speech production and phonological processing improve following cochlear implantation [29,43,56], likely reflecting the benefits of restored auditory input on phonological representations. One way of assessing the effects of changes in phonological representations after cochlear implantation through speech production is by identifying the extent to which CI users produce phonetic contrasts between similar-sounding words or phonemes, which can entail measuring either the acoustic aspects or the subjective intelligibility of speech produced by CI users [28,29,30,31,43,56,57]. Further, addressing the relationship between these measures of production to measures of perception can provide insight into the production–perception link in CI users.

Acoustic measures of vowel production, like vowel space area and first and second formant (F1 and F2) frequency measurements, have been used in both cross-sectional and longitudinal studies to quantify speech production in CI users [31,56,57,58]. However, few studies have compared acoustic measures of speech production to speech perception in CI users. Ref. [59] collected longitudinal vowel production data for four CI users in addition to performance on a closed-set vowel identification (perception) task. The authors of that study did not directly compare production and perception measures to one another but found that increases in vowel identification accuracy over about 18 months post-activation were accompanied by global increases in F2 across all vowels (corresponding to fronting of the acoustic–phonetic vowel space). These results demonstrate that changes in production and perception following implantation tend to trend together, at least in the first 18 months.

One acoustic measure of vowel production that may be related to subjective speech intelligibility entails measuring of the overall size of the acoustic–phonetic vowel space to characterize both phonological representation of contrast between vowels and the resulting intelligibility of vowel productions. Auditory deprivation can lead to overall compression of the vowel space, resulting in vowel productions that are ambiguous or confusable with one another [60,61,62,63]. Vowel dispersion is an acoustic metric used to measure relative expansion or compression of the vowel space and is quantified as the Euclidian distance from a vowel-dependent midpoint of a talker’s vowel space to the acoustic realization of a vowel in the F1 × F2 dimension, e.g., [10,50,64,65,66]. Talkers who produce more dispersed vowels are more intelligible than those who produce less dispersed vowels [64], at least as assessed using vowel dispersion measures from vowels in running sentences and measuring intelligibility as the number of words in a sentence that a listener correctly reports back. These findings suggest that vowel dispersion could serve as an acoustic predictor of speech intelligibility.

Restoration of auditory feedback provided by the CI in post-lingually deafened adults can allow CI users to enhance the acoustic–phonetic contrast between speech sounds, and it has been assumed that this contrast enhancement can facilitate the intelligibility of their speech [28,29,56,57]; for reviews, see [67,68]. In one study that examined changes in speech production longitudinally following cochlear implantation, Ref. [57] assessed acoustic and perceptual measures of speech production in post-lingually deafened adults before cochlear implantation and at 1 month, 6 months, and 2 years post-CI activation. They found acoustic changes in speech production up until 2 years post activation, such that the CI users’ speech became more acoustically similar to that of their NH peers over time. In addition, perceptual ratings of the quality of the CI users’ speech indicated that their speech became more subjectively intelligible over the 2-year period following CI activation, as rated by trained clinicians. These results suggest that the restoration of auditory input via CI and experience with the signal delivered by the CI can yield improvements in both speech perception and production relative to performance pre-implant.

Due to the substantial variability observed in speech perception outcomes post-implantation, several studies have sought to identify potential pre-implant predictors of post-implant performance. For example, in pre-lingually deafened adults, Refs. [69,70] found that CI users with more subjectively intelligible speech prior to cochlear implantation demonstrated stronger speech recognition outcomes post-implantation than CI users with less intelligible speech pre-implantation. These findings suggest that aspects of speech production are related to speech perception in CI users, even when speech production is assessed prior to implantation. These results potentially reflect a common underlying cognitive–linguistic mechanism—phonological processing—that both influences the intelligibility of speech pre-implant and shapes the success with which new CI users can adapt to the degraded signal to recognize and perceive speech post-implant. In addition, these results highlight the potential clinical utility of using intelligibility of speech production as both a predictive measure of clinical speech recognition outcomes and as a standalone clinical outcome measure. Given that both speech production and perception draw on phonological representations, investigating the relationship between speech production, speech perception, and phonological processing may provide a clearer window into the mechanisms that underlie variability in CI production and perception outcomes.

### 1.4. The Current Study

The purpose of this exploratory study is to establish the relationship between individual differences in speech production and speech perception in experienced adult CI users. This cross-sectional study focused on stable patterns of speech production and perception among CI users with greater than 2 years of CI use, rather than longitudinal change, to establish preliminary relationships among measures. We analyzed both subjective perceptual ratings of speech intelligibility and acoustic features of vowel distinctiveness using isolated words elicited from 15 CI users in a word reading task. The primary goal of this study was to establish the relationships among the subjective intelligibility of a CI user’s speech, their speech recognition accuracy, and an indirect measure of phonological processing. As a secondary goal, we determined whether an acoustic measure of clarity of speech production, vowel dispersion, is associated with both speech recognition accuracy and phonological processing. Finally, we sought to determine whether vowel dispersion is a reliable predictor of the subjective intelligibility ratings of a CI user’s speech. We hypothesized, first, that subjective ratings of CI speech intelligibility are associated with both speech recognition and phonological processing. We further hypothesized that vowel dispersion is associated with both speech recognition and phonological processing. Finally, we expected that a higher degree of vowel dispersion would lead to more subjectively intelligible utterances than vowels produced with less dispersion. These findings will serve to establish preliminary evidence of relationships among measures of speech production, perception, and phonological processing in adult CI users, which could motivate future work to identify the utility of these metrics as predictive and explanatory clinical metrics of performance with a CI. In turn, these findings could provide clinicians with feasible metrics by which to assess CI speech production and predict and explain variability in perception outcomes.

## 2. Materials and Methods

### 2.1. Participants: Cochlear Implant Users

Fifteen adult CI users participated in the current study. All CI users were peri- or post-lingually deafened, defined as having an onset of HL at or after 12 years of age. CI users were identical to those described in [50] and formed a subset of participants from [51]. CI users ranged in age from 24 to 76 years (mean age = 58.5 years, SD = 13.8). Ten participants were female and five were male. Seven of the CI users had a bimodal listening configuration, with one CI and a hearing aid in the contralateral, non-implanted ear. Five participants were bilateral CI users, and three were unilateral CI users (one CI and no hearing aid in the contralateral ear). The mean duration of deafness, defined as the years between self-reported onset of HL and age at first CI, was 30.1 years (SD = 15.2). Duration of deafness information was unavailable for 4 of the 15 CI participants, who were unable to report when their HL began. A summary of demographic information for CI users is included in Table 1. During testing, CI users wore their own devices (including contralateral hearing aids, if applicable), set to their everyday clinical settings to most closely replicate real-world communicative conditions. All participants were native, monolingual speakers of American English and reported no history of speech or cognitive disorders. Participants were recruited through the Department of Otolaryngology at The Ohio State University Wexner Medical Center, through The Ohio State University, and from the surrounding Columbus, Ohio metropolitan area. This study was approved by the Institutional Review Board at The Ohio State University. CI users completed all tasks in the laboratory in an audio booth or sound-treated testing room.

### 2.2. Participants: Normal Hearing Listeners

Forty-seven adults with self-reported NH were recruited to rate the subjective intelligibility of the CI users’ speech. All participants were recruited and participated online using the Prolific recruitment service and gorilla.sc experimental platform [71] using their own desktop or laptop devices and headphones. Prior to participation, NH participants completed a headphone screener [72] and were excluded from analysis if results from these screeners suggested that they were not wearing headphones (i.e., a score of <5 out of 6 on the headphone screener). Ten participants were excluded in this way, resulting in a total of 37 participants retained for the current analysis. These 37 participants ranged in age from 18 to 60 years (mean age 29.4) and included 20 women and 17 men. All participants were native speakers of American English with self-reported NH and no speech or language disorders.

### 2.3. Speech Production: Word Reading and Acoustic Analysis

#### 2.3.1. Materials

Materials for the CI word reading task were the same as those described in [50]. Each CI participant read 240 monosyllabic CVC words that varied in neighborhood density and lexical frequency. Neighborhood density was operationalized as the number of words differing from a target word by the addition, deletion, or substitution of a single phoneme [8] and was obtained for each word using the University of Kansas Similarity Neighborhood Calculator or the Hoosier Mental Lexicon [73]. Lexical frequency was operationalized as the log of the contextual diversity in the SUBTLEX_US_ database, which derives word frequency information from movie subtitles [74,75]. Contextual diversity is defined within SUBTLEX_US_ as the count of movies where a target word is identified within its subtitles.

For the current study, a subset of 84 of these 240 words were selected for analysis, which included point vowels [æ, ɑ, i, u]. Point vowels have F1 × F2 values that represent the extreme points of the vowel quadrilateral, and previous studies found that these vowels yielded differences in vowel dispersion across vowel categories [10,66].

#### 2.3.2. Procedure

CI users were seated with the experimenter in a quiet room. Words were presented visually, one at a time, on a computer screen, using the gorilla.sc experimental platform. Participants were instructed to read each word aloud naturally as it appeared on the screen. The experiment advanced automatically to the following word after 2500 ms. Word order was randomized for each participant. Participant responses were digitally recorded using a Shure cardioid condenser microphone with a 44.1 kHz sampling rate and 16-bit resolution. The total duration of the task was about 15 min.

#### 2.3.3. Acoustic Analysis

In order to derive an acoustic metric of vowel distinctiveness for each token and each talker, a measure of dispersion from the center of each talker’s vowel space was calculated for each vowel token. Each recording was acoustically analyzed to identify F1 and F2 for each vowel in each CVC word. First, vowels in audio recordings were annotated using a forced aligner (MAUS; [76]) and manually verified by the first author in Praat [77]. The temporal midpoint of each annotated vowel was identified using Parselmouth, a Python library (3.9.16) that interfaces with Praat [78], and formant values were estimated at the measured midpoint of each vowel. For analysis, formant values were converted from Hz to Bark [79] following procedures in similar acoustic analyses [10,50,65,66]. Words that were unable to be acoustically analyzed were excluded, including words that participants misread (e.g., “ran” instead of “rune”) and words that were otherwise interrupted (e.g., by a cough). The total number of words per each of the 15 CI talkers included in the current analysis is provided in Table 2, shown separately by vowel category.

In order to derive a measure of dispersion from the center of each talker’s vowel space for each vowel, the estimated center of each talker’s vowel space was calculated separately for each of the 15 CI users. Following previous studies that have calculated vowel dispersion [65,80,81], the center of the vowel space was defined as the grand mean of the F1 and F2 values for /i/ and /ɑ/. However, given that the vowels used in this analysis were not evenly distributed around the vowel space and words used for analysis did not include an equal number of vowels for each talker, the resulting acoustic centers of the vowel space are dependent on the vowels used in the task and analysis and do not necessarily represent a true articulatory or perceptual midpoint. Vowel dispersion was calculated and defined as the Euclidian distance in the F1 × F2 Bark space from a given instance of a vowel to the center of that talker’s acoustic vowel space.

### 2.4. Intelligibility Ratings of Cochlear Implant Users’ Speech

#### 2.4.1. Materials

Recordings of words spoken by the 15 CI participants were used as stimulus materials for NH raters. Words that included point vowels [æ, ɑ, i, u] were included in addition to words that included additional vowels, which were examined separately as part of a larger study (only words with point vowels were included in the current analysis). Across all vowels, words that were unable to be acoustically analyzed due to mispronunciations or misreadings were excluded from the auditory stimuli presented to the NH raters. Recordings were trimmed to remove silence before and after each utterance and normalized to the same RMS level prior to presentation.

To ensure that each word spoken by each CI user was rated by multiple listeners, 10 lists of between 352 to 355 stimuli were created. Each list included between 16 and 33 utterances from each of the 15 talkers, determined based on the total number of available tokens from each CI user. Lists were pseudo-randomly assigned to each of the 37 NH raters. Each of the 1068 tokens included in the current analysis were rated between 4 and 8 times, with a mean of 5.2 ratings given for each token.

#### 2.4.2. Procedure

NH participants completed the experiment on the gorilla.sc platform using their own desktop or laptop devices and headphones. They were asked to sit in a quiet room for the duration of the experiment. Listeners completed a headphone screener prior to participation. The online headphone screener consisted of a three-alternative forced-choice psychophysical task in which listeners heard three 1000 ms sequences of white noise and were asked to identify the interval that differed from the other two. In two of these intervals, the white noise bursts delivered to each ear were identical. In one of these intervals, one of the white noise bursts was shifted 180 degrees in phase relative to the noise presented to the opposite ear, yielding the percept of a faint tone embedded in noise. Critically, this percept is only present when the two signals are presented dichotically combined over headphones, so participants listening over loudspeakers are not likely to pass the screener [72]. For the purpose of the current study, participants who scored < 5 correct answers out of 6 were considered to have failed the screener and not included in the current analysis. Although not designed to screen for HL or identify background noise, listeners with hearing loss and/or listeners who are participating in a noisy environment may also fail this screener [82].

For the main intelligibility rating task, listeners were pseudo-randomly assigned to 1 of 10 possible lists of auditory stimuli, described above. Each list contained words spoken by each of the 15 talkers. Following five practice trials, listeners were presented with one word at a time and asked to rate how understandable that word was using a sliding scale with endpoints “not understandable at all” to “very understandable” visible on each side of the sliding scale for the duration of the task. Points on the scale were numerically coded as 1 (not understandable) to 100 (very understandable), though these numbers were not visible to participants. Participants were instructed to ignore differences in audio quality or sound quality across tokens and focus on how understandable or intelligible each word was. They were informed that all words were real words in the English language. Each auditory stimulus was preceded by a 500 ms fixation cross on the computer screen. Tokens were fully randomized within each list. Rating responses were recorded as numbers between 1 and 100 for analysis.

### 2.5. Speech Perception Tasks (Outcome Measures)

#### 2.5.1. Phonological Processing: Word and Nonword Reading Efficiency

The Test of Word Reading Efficiency, Second Edition (TOWRE-2; [83]), was used as an indirect measure of phonological processing. This measure assesses single-word reading accuracy and fluency in the absence of sentence context. Participants completed two subsets of this test, including rapid real-world reading and nonsense word reading. Participants were asked to read as many real words as possible in 45 seconds from a 108-word list and as many nonsense words as possible from a 66-nonword list. Form lists A were used. Video recordings were used to transcribe responses by two trained scorers who had previously attained 95% agreement with an established reliable scorer of TOWRE-2. Each participant’s TOWRE-2 was scored by one primary and one secondary scorer, and scores from the primary scorer were used for analysis. No data were omitted. For analysis, one total score was computed per participant by summing the total number of real words and nonwords correctly read out loud. We chose to analyze this aggregated TOWRE-2 total score, rather than analyze words and nonwords separately, based on findings that this total score is associated with three different measures of sentence recognition accuracy in CI users in both cross-sectional and longitudinal studies [51,84]. In contrast, the separate associations between word reading efficiency and speech recognition and nonword reading efficiency and speech recognition are inconsistent across these studies.

#### 2.5.2. Sentence Recognition Accuracy

Sentence recognition in quiet was assessed using the Perceptually Robust English Sentence Test Open-Set (PRESTO; [85]). PRESTO maximizes talker variability across sentence materials by including sentences produced by multiple talkers with different genders, regional dialects, and speaking rates. PRESTO is similar to sentence materials used for traditional clinical testing but was chosen over these traditional clinical measures in order to be relatively unfamiliar to participants. Original PRESTO sentence lists were balanced for talker gender, lexical frequency of sentence keywords, and familiarity of sentence keywords, with no repeated talkers. For this study, participants were presented with 36 sentences (PRESTO lists 7 and 8), with the first 2 sentences from list 7 used as practice. Each PRESTO list included 18 sentences, ranging from 5 to 10 words per sentence. The number of keywords per sentence ranged from 3 to 5, but each list included a total of 76 keywords. Auditory stimuli were presented at 68 dB SPL via a Roland MA-12C loudspeaker placed 1m directly in front of the participant’s head. Participants heard one sentence at a time over the loudspeaker and were asked to repeat back each sentence out loud and to guess if unsure. A single accuracy score was calculated per participant and defined as the percentage of sentence keywords correctly identified from each of the PRESTO sentences not used as practice, with a total of 143 total possible keywords. As with TOWRE-2, each participant’s PRESTO keyword recognition accuracy was scored by one primary and one secondary scorer who had previously been trained until they reached 95% agreement with an established reliable scorer and with each other. Scores from the primary scorer were used for analysis, and no data were omitted.

### 2.6. Data Analysis

To address the objectives of this study, a series of Pearson’s correlations were conducted to examine the associations between several pairs of variables. The distribution of data for each variable was approximately normal, based on visual inspection of histograms and visual inspection of output from the qqnorm() and qqline() functions in R (4.5.0). First, Pearson’s correlation analyses were conducted to establish the associations between subjective ratings of the intelligibility of CI users’ speech and speech recognition accuracy (PRESTO) and between intelligibility ratings and phonological processing efficiency (TOWRE-2). A second series of Pearson’s correlations were conducted to identify the relationship between vowel dispersion and speech recognition accuracy (PRESTO) and vowel dispersion and phonological processing efficiency (TOWRE-2). Finally, to assess the validity of vowel dispersion as a metric of the subjective intelligibility of a talker’s speech, a linear mixed-effects regression model was used to predict subjective ratings of CI users’ speech intelligibility by NH raters from vowel dispersion. Mixed-effects modeling was chosen for the final analysis because repeated measures were available for both vowel dispersion and intelligibility ratings, whereas PRESTO and TOWRE-2 data were collected as part of a larger study, and therefore, repeated trial-by-trial measures were unavailable.

**Figure 1:**
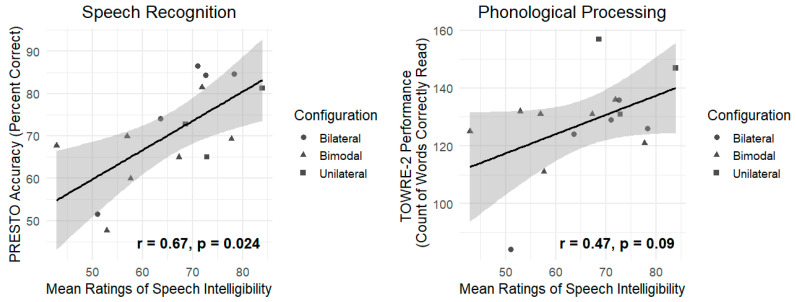
Relationships between intelligibility ratings and PRESTO accuracy (**left panel**) and intelligibility ratings and TOWRE-2 number of words read (**right panel**). Solid lines represent the best-fit linear regression line and shading represents the 95% confidence interval of the fitted regression line. Coefficients and associated *p*-values from Pearson’s correlations are provided. Participants’ listening configurations are denoted by the shape of each point.

**Figure 2:**
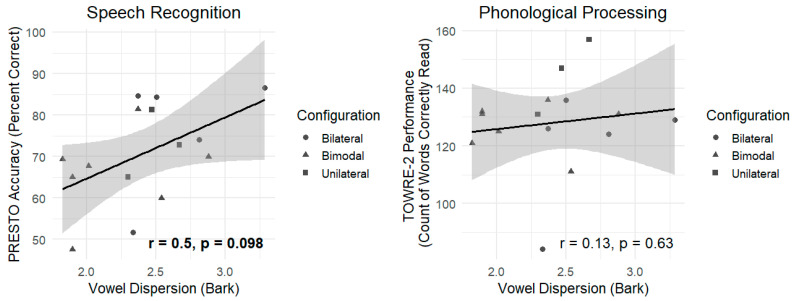
Relationships between vowel dispersion and PRESTO accuracy (**left panel**) and vowel dispersion and TOWRE-2 number of words read (**right panel**). Solid lines represent the best-fit linear regression line and shading represents the 95% confidence interval of the fitted regression line. Coefficients and associated *p*-values from Pearson’s correlations are provided. Participants’ listening configurations are denoted by the shape of each point.

For correlational analyses, predictor measures (intelligibility ratings and vowel dispersion) were first aggregated by talker, vowel, and lexical difficulty to account for inherent differences in subjective ratings of speech clarity and vowel dispersion based on vowel category and lexical difficulty [10,66,86] and because the number of tokens used for analysis differed across vowel category and lexical difficulty by talker. Then, for each talker, average intelligibility ratings and average vowel dispersion were calculated over these aggregated data. For all correlational analyses, the false discovery rate (FDR) correction was used to correct for multiple comparisons, and corrected *p*-values are reported. Finally, we note that our sample of participants includes bilateral CI users (n = 5), bimodal CI users (n = 7), and unilateral CI users (n = 3). Due to the small sample size, we cannot draw conclusions about differences across listening configurations. However, we have plotted the data in Figure 1 and Figure 2 using different shapes for each listening configuration to facilitate visualization of potential group-level trends.

## 3. Results

A summary of mean intelligibility ratings, mean vowel dispersion, PRESTO scores (sentence recognition accuracy), and TOWRE-2 scores (phonological processing efficiency) is provided in Table 3 for each CI user. Across all CI users, the mean intelligibility, as evaluated by NH raters, was 65.9 (range 42.9 to 84.0). Mean vowel dispersion was 2.41 Bark (range 1.8 to 3.3). Mean PRESTO accuracy was 70.8% keywords correct (range 47.6 to 86.6). Finally, the mean TOWRE-2 total score was 128.1 total combined words and nonwords correctly read out loud (range 84 to 157).

### 3.1. The Association Between Intelligibility Ratings and Speech Perception Outcomes

Pearson’s correlation was carried out to establish the association between subjective ratings of the intelligibility ratings of CI users’ speech and CI users’ PRESTO scores of sentence recognition. Results are displayed in Table 4 and the left panel of Figure 1. Intelligibility ratings were moderately to strongly positively correlated with PRESTO accuracy (r = 0.67, *p* = 0.024), revealing that CI users who were rated as having more intelligible speech performed more accurately on sentence recognition than CI users with less intelligible speech.

Pearson’s correlation was carried out to establish the association between subjective ratings of the intelligibility of CI users’ speech and overall TOWRE-2 scores. Results are displayed in Table 4 and the right panel of Figure 1. The relationship was marginally significant: intelligibility ratings were moderately positively correlated with TOWRE-2 performance (r = 0.47, *p* = 0.09), potentially revealing that CI users who were rated as having more intelligible speech demonstrated somewhat more efficient word reading and phonological processing than CI users with less intelligible speech, though this relationship did not reach statistical significance at an alpha of 0.05.

### 3.2. The Association Between Vowel Dispersion and Speech Perception Outcomes

Pearson’s correlation was carried out to establish the association between vowel dispersion and PRESTO scores. Results are displayed in Table 4 and the left panel of Figure 2. The relationship was marginally significant: vowel dispersion was moderately positively correlated with PRESTO accuracy (r = 0.50, *p* = 0.098), potentially revealing that CI users who produced more distinct vowels demonstrated somewhat more accurate sentence recognition than CI users who produced more ambiguous vowels, though this relationship did not reach statistical significance at an alpha of 0.05.

Pearson’s correlation was carried out to establish the association between vowel dispersion and overall TOWRE-2 scores. Results are displayed in Table 4 and the right panel of Figure 2. No clear relationship was observed (r = 0.13, *p* = 0.63), indicating that acoustic distinctness of vowel production does not appear to be associated with this measure of phonological processing.

### 3.3. The Relationship Between the Two Measures of Speech Production

A linear mixed-effects regression model was used to identify the impact of vowel dispersion on subjective intelligibility ratings. A maximal, data-driven random-effects structure was initially specified [87] and random slopes were removed until the model converged. Statistical significance was assessed using the Satterthwaite approximation of degrees of freedom for F-statistics, implemented using the anova function within the lmerTest package in R [88,89]. The dependent measure in the current analysis was listeners’ intelligibility ratings and was predicted from vowel dispersion. Based on evidence that words’ lexical frequency and neighborhood density influence subjective ratings of speech clarity [86], both lexical frequency and neighborhood density were included as covariates. All variables and covariates were continuous. In the final model, random intercepts for talker (CI user), listener (NH rater), and item (word) were included. For all measures, an alpha of 0.05 was set. A summary of model output is included in Table 5.

The results revealed no main effect of vowel dispersion on intelligibility ratings [F(1,4745) = 0.47, n.s.], suggesting that subjective ratings of speech intelligibility did not depend on the degree of dispersion with which vowels were produced. The lexical frequency covariate reached significance [F(1,86.3) = 7.04, *p* = 0.009], revealing that words with a higher frequency of occurrence in spoken language were rated as more intelligible than words with a lower frequency of occurrence. The neighborhood density covariate did not reach significance [F(1,84.3) < 0.001, n.s.].

## 4. Discussion

The primary goal of this exploratory study was to establish the relationships among the subjective intelligibility of a CI user’s speech and their performance on measures of speech recognition and phonological processing. As a secondary goal, we determined whether vowel dispersion, an acoustic metric of speech production, was associated with speech recognition performance and phonological processing. Finally, we determined whether vowel dispersion is a reliable predictor of the subjective intelligibility ratings of a CI user’s speech. We hypothesized, first, that subjective ratings of speech intelligibility are associated with both speech recognition and phonological processing. Similarly, we expected that vowel dispersion is also associated with speech recognition and phonological processing. Finally, we predicted that words with more dispersed vowels would be rated as more subjectively intelligible than words with less dispersed vowels. Due in part to the small sample size of CI users in the current study (n = 15), this exploratory study is intended to establish potential relationships among cognitive–linguistic factors to motivate future investigation. Accordingly, in the following sections, we will suggest possible factors underlying the observed results and suggest avenues for future work to delineate the relationships between speech production, phonological processing, and speech perception in adult CI users.

### 4.1. Subjective Ratings of Speech Intelligibility Are Associated with Speech Recognition

In support of our primary hypothesis, the results from the correlational analyses demonstrated a significant, positive, moderate-to-strong association between subjective intelligibility ratings of CI users’ speech and sentence recognition accuracy. Specifically, we found that CI users who were rated as having more intelligible speech also demonstrated more accurate sentence recognition than CI users who had less intelligible speech. Though relatively few studies have examined the intelligibility of the speech of post-lingually deafened CI users, these findings are consistent with those of longitudinal studies examining changes in the intelligibility of CI users’ speech utterances pre- and post-implant [30,57,90]. For example, Ref. [57] found that improvements in speech recognition accuracy over the first two years after CI activation were accompanied by increases in the subjective clarity of speech utterances of the participants, and these improvements appeared to follow similar trajectories. Examining changes in intelligibility over time, particularly for post-lingually deafened adults, is limited by the fact that many participants have high baseline (pre-implant) intelligibility (often reflected by the choice to assess the intelligibility of these talkers by embedding their utterances in background noise to avoid ceiling effects; e.g., [30,90]). Therefore, it may be difficult to assess improvement in speech production post-implant for talkers with relatively high baseline intelligibility pre-implant. However, despite high intelligibility among some post-lingually deafened adults, several studies have noted substantial variability in intelligibility pre-implant [30,91]. This variability suggests that not all post-lingually deafened adults consistently produce highly intelligible speech. This variability was also present among the current sample of 15 CI users who had at least 2 years of CI experience: mean intelligibility ratings ranged from 42.9 to 84.0 per talker, averaged across NH raters and stimulus words (see Table 3).

Understanding the time course of improvements in perception vs. production in adult CI users can provide insight into mechanisms underlying these improvements (for example, whether improvements in one drive improvements in the other). Evidence from the second language (L2) acquisition literature in adults may shed light on these changes. Some studies that have examined L2 acquisition patterns over a short timescale early in the acquisition process have found that gains in speech perception precede speech production improvements [92]. However, at later stages of learning, changes in production may precede changes in perception [40]. Further, although evidence is mixed, there is some evidence that training L2 learners in production may yield improvements in perception under certain conditions [93,94]. These results may suggest that the time course of acquisition of perception and production skills differ, or potentially that improvements in one skill drive improvements in the other at different stages of learning. To the extent that L2 acquisition shares cognitive and linguistic resources with the process of re-mapping sounds delivered through a CI to existing phonological representations, this evidence collectively suggests a complex link between the development of speech recognition and speech production. Further, these findings demonstrate that this relationship may change over time, as with more experienced L2 learners or more experienced CI users.

Methodological choices in assessing intelligibility of CI users’ speech may have facilitated our observation of the association between speech intelligibility ratings and speech recognition scores. Studies that have observed small or no improvements in the intelligibility of CI users’ speech from pre- to post-implant primarily had listeners perform closed- or open-set speech recognition or shadowing tasks in background noise [30,43,90]. Accuracy in these tasks is often high, given the relatively good intelligibility of the speech of post-lingually deafened CI users [90]. However, tasks eliciting a more subjective rating of speech intelligibility (sometimes referred to as clarity or comprehensibility [86]; or voice quality [57]) typically yield a wide range of responses both within and across NH raters, even when the speech is objectively intelligible (i.e., when listeners would be able to accurately identify the word they were presented with). Our preliminary results, along with those from studies that have used similar techniques to elicit subjective ratings of intelligibility, provide support for the use of these ratings to assess perceptually meaningful aspects of CI users’ speech that may not be captured by traditional tests of recognition accuracy of these utterances.

Our results also revealed a nonsignificant but positive association between subjective ratings of speech intelligibility and TOWRE-2 scores. While not statistically significant, this trend toward a positive association may tentatively suggest a relationship between speech intelligibility and phonological processing, such that CI users with speech that was rated as more subjectively intelligible demonstrated more efficient phonological processing than CI users with less intelligible speech. The absence of a more robust relationship between intelligibility ratings and TOWRE-2 scores may reflect limitations in the sensitivity of the TOWRE-2 task as an indirect measure of phonological processing and representations. This task may not fully capture the specific phonological mechanisms relevant to speech intelligibility. Further, nonauditory cognitive load and literary factors may also influence performance on TOWRE-2, potentially impacting our results. We did not assess the relationship between phonological processing and speech recognition accuracy in the current study, but previous work has revealed that post-lingually deafened CI users with better word reading efficiency (operationalized as higher total word + nonword TOWRE-2 reading fluency scores, as used in the current study) also demonstrated higher PRESTO sentence recognition accuracy [51,84]. An exhaustive investigation of the extent to which phonological processing mediates the relationship between speech perception and production in adult CI users is beyond the scope of this paper. However, based on evidence from NH adults that phonological processing influences speech perception and speech production [9,10], these results motivate further study into the role of phonological processing in speech communication outcomes among adult CI users.

The observed relationships between speech perception, speech production, and phonological processing are consistent with several existing models of speech processing. To the extent that phonological representations are entailed in the speech sound map described by the DIVA model of speech production [33,35], DIVA posits that auditory and perceptual information derived from exposure to spoken language contributes to the formation and maintenance of the speech sound map. This relationship between mapping auditory input onto phonological representations is further corroborated by the Ease of Language Understanding (ELU) model [95], which posits that speech recognition is facilitated by robust mappings between auditory input and phonological representations. Taken together, these models support a link between speech recognition and phonological representation, and previous studies have provided experimental evidence in support of this link in adult CI users [20,21,22,24,25,26,47,51]. Importantly, this relationship exists despite the degraded auditory signal delivered by the CI, potentially suggesting either that phonological representations formed prior to hearing loss are able to be recovered via auditory input delivered by the CI, or that the modification of phonological representations is possible with the novel input provided by the CI. 

The relationship between phonological processing and intelligibility of speech production is less well studied, particularly among post-lingually deafened adult CI users. However, DIVA proposes direct and indirect links between speech sound maps (similar to phonological representations) and speech production via feedback control of the auditory and somatosensory systems. Experimental evidence in support of this relationship comes from neural imaging and behavioral studies alike [31,96,97]. In addition, studies investigating how phonological representations influence speech production, both in NH adults and adult CI users, have shown that talkers can use their knowledge of phonological contrast (i.e., differences in speech sounds) to enhance or reduce acoustic–phonetic realizations of their speech [10,50,65,66,80,81]. For example, evidence from vowel production studies suggests that talkers hyperarticulate vowels in short words when there are many similar-sounding words in the lexicon of that talker, presumably in order to enhance phonetic contrast between the target utterance and other similar-sounding words [10,66,80]. This phonetic realization of underlying phonological contrast supports the notion that phonological processing and speech production are linked, and preliminary evidence of these speech production patterns has been observed in adult CI users [50]. Phonetic contrast enhancement is one acoustic marker of clear speech and can therefore enhance the intelligibility of speech utterances by disambiguating between a target utterance and similar sounds in a talker’s or listener’s lexicon [98,99]. Evidence from this literature somewhat contradicts the non-significant association we observed between intelligibility ratings and phonological processing in the current study, though our measure of phonological processing (TOWRE-2) differs from the measures of phonetic contrast used in this literature. Therefore, future investigations should assess the validity and sensitivity of these different measures in assessing phonological processing and representations.

### 4.2. Vowel Dispersion Is Not Associated with Speech Recognition, Phonological Processing, or Intelligibility Ratings

Given the evidence that phonetic contrast enhancement is reflective of underlying phonological representations in NH adults [10] and that acoustic measures of vowel distinctiveness are associated with speech intelligibility [64,99], we investigated the extent to which vowel dispersion, an acoustic measure of vowel distinctiveness, was associated with speech recognition accuracy and phonological processing in CI users. The results revealed a non-significant but positive association between vowel dispersion and sentence recognition accuracy, such that talkers who produced vowels further from the center of their vowel space tended to demonstrate higher sentence recognition accuracy than talkers who produced vowels closer to the center of their vowel space. The absence of a significant association between vowel dispersion and PRESTO accuracy is somewhat inconsistent with our finding that CI users with more intelligible speech demonstrate higher sentence recognition accuracy than CI users with less intelligible speech. This discrepancy could suggest that intelligibility ratings and vowel dispersion reflect different underlying processes in speech production. Alternatively, it is possible that our measure of vowel dispersion is not sensitive enough or our study is not adequately powered to detect a reliable relationship between these measures. 

The absence of a relationship between vowel dispersion and phonological processing raises additional questions about the relationship between vowel dispersion and intelligibility ratings. Given previous work that has shown a relationship between vowel dispersion and speech intelligibility [64] and that clear speech, which is typically more intelligible than conversational speech, is characterized in part by high degrees of vowel dispersion and large vowel spaces [99,100,101], we expected that enhanced vowel dispersion would be a feature of intelligible speech and would therefore both be related to sentence recognition and phonological processing.

To assess our assumption that vowel dispersion and intelligibility are related, we conducted a repeated measures analysis to assess whether more dispersed vowels were associated with higher subjective intelligibility ratings than less dispersed vowels. The results revealed that vowel dispersion did not predict intelligibility ratings, suggesting that the dispersion, or distinctiveness, of a given vowel in a CVC word did not contribute to how intelligible listeners rated the word as. This finding somewhat conflicts with that of [64], who found that keywords in sentences produced by talkers with more dispersed vowels were more accurately transcribed than keywords produced by talkers with less dispersed vowels. However, the materials from which vowel dispersion were derived differed across our studies: [64] calculated dispersion based on vowels from mono- and multisyllabic words in sentences, whereas dispersion in our study was calculated based only on vowels in isolated monosyllabic words. Different patterns of vowel dispersion have been observed in vowels in words extracted from conversational speech compared to words read in isolation [102]. Therefore, the patterns of vowel dispersion observed in [64] and in the current study may have differed due to the more conversational nature of words extracted from sentences compared to words read in isolation. However, further work is needed to delineate the impact of these potential differences in vowel dispersion on intelligibility.

A second reason why we may have failed to find evidence of a relationship between vowel dispersion and intelligibility ratings is due to potential differences between subjective intelligibility ratings, as employed in the current study, and speech recognition accuracy, as used in [64]. While these subjective ratings can be informative, particularly when intelligibility is high [57,69,70,86,90], they may recruit different auditory or cognitive processes than those required for speech recognition (i.e., transcription or shadowing) tasks. Evidence from NH L2 learners suggests that comprehensibility ratings of speech from nonnative talkers (similar to intelligibility ratings in the current study) are somewhat dissociated from speech recognition accuracy, and the authors speculate that comprehensibility ratings could reflect something similar to the effort expended while listening to an utterance rather than how intelligible the utterance was [103,104].

Finally, listeners may have used other properties of speech beyond just vowel dispersion when rating speech intelligibility. Vowel dispersion characterizes the acoustic properties of vowels within words but does not reflect the surrounding consonantal context or suprasegmental factors, like articulation rate, which are known to influence perceived intelligibility [105]. Therefore, the absence of a strong relationship between vowel dispersion and intelligibility ratings may indicate that, at least for some listeners, factors beyond acoustic properties of the vowel may influence perceived intelligibility of an utterance. Broader speech cues may therefore be more relevant for identifying CI users with poorer speech recognition outcomes, though further research is needed to evaluate the impact of these cues on intelligibility ratings.

One factor that may explain the lack of relationship between vowel dispersion and phonological processing is the way that vowel dispersion patterns are interpreted in the literature with respect to phonological representations. Specifically, vowel dispersion is often used as a metric by which to quantify phonetic contrast, assumed to reflect the structure of underlying phonological representations [10,50,65,66,80,81,102]. However, to measure contrast, there are typically least two factors that are being contrasted with one another. For example, in the studies listed above, vowel dispersion was compared across vowels in words that were lexically easy (i.e., occur often in spoken language, have few similar-sounding words, and tend to be intelligible) vs. lexically hard (i.e., occur infrequently, have many similar-sounding words, and are less likely to be intelligible). Therefore, the overall measure of vowel dispersion used in the current study may be less representative of phonological processing than a comparison of vowel dispersion between vowels across different lexical contexts, and this consequently may further explain the lack of a relationship between vowel dispersion and TOWRE-2 scores in the current study. However, other studies have used vowel dispersion or related measures to assess how distinct specific vowel utterances or utterances of vowel categories are to one another in adult CI users [31,32,106]. Accordingly, future analyses should pair acoustic measures of vowel distinctiveness with measures of the acoustic variability in vowel production (as in [106]), which could also account for inherent variability in formant measurements (especially in CI speech), or they should compare instances of vowel dispersion across particular words or vowel categories in order to more comprehensively characterize the acoustic markers of phonological contrast in adult CI users.

### 4.3. Limitations

This study is exploratory and is intended to establish the feasibility subjective ratings of speech intelligibility as a potential predictor of speech recognition performance among post-lingually deafened adult CI users. Due to the small sample size (n = 15), which limits statistical power and generalizability, the cross-sectional nature of the study, and analyses that are primarily correlational in nature, future work is necessary to determine the causality of the associations observed and to identify potential mechanisms that mediate or moderate these relationships. For instance, examining separate relationships between measures of speech production and word and nonword reading efficiency (rather than using an aggregated TOWRE-2 score) may provide additional insight into the relationship between speech production and phonological processing. In addition, multiple demographic, cognitive, audiological, and linguistic factors are known to contribute to measures used in the current study, which we did not examine in detail. For example, participants’ listening configurations—specifically, whether they receive acoustic auditory input through a hearing aid in addition to a CI—may influence the quality of auditory feedback they receive, but no clear trends based on listening configuration were apparent in the current study. In the following section, we briefly review some factors that may contribute to individual differences in measures of speech production and perception. The contribution of these factors to outcome measures should be more thoroughly evaluated before the clinical utility of intelligibility measures can be determined.

### 4.4. Future Directions: Other Factors That May Influence Speech Production and Intelligibility Ratings

Due to our small sample size, we were unable to conduct an exhaustive analysis of individual differences among CI users and NH raters and how they contributed to intelligibility ratings. However, some factors that may impact speech intelligibility are outlined below as directions for future analyses. Briefly, these factors encompass those related to properties of the stimuli, audiological and demographic characteristics of the CI users, and characteristics of the NH raters.

Properties of the stimuli used in this and other studies have been shown to influence subjective ratings of clarity and intelligibility. In the current study, we included lexical frequency as a covariate in our analysis predicting intelligibility ratings from vowel dispersion. The lexical frequency covariate reached significance, expanding upon previous findings that words with a higher frequency of occurrence in spoken language are rated as more clear or more intelligible than words with a lower frequency of occurrence [86]. In addition, while not directly assessed in the current study, previous studies have determined that speaking rate influences comprehensibility ratings (similar to intelligibility ratings in this study), such that talkers with very fast or very slow speaking rates were rated as less comprehensible than other talkers [105]. Therefore, lexical factors and speaking rate should be accounted for in future studies assessing intelligibility ratings.

Audiological, demographic, and cognitive factors of the talkers who produce stimuli may also be associated with individual differences in outcomes. For example, duration of deafness prior to cochlear implantation has been found in some studies to be negatively correlated with intelligibility [91], and some studies have found that longer durations of deafness are associated with more degraded phonological representations than those with shorter durations of deafness [22,23,49], though challenges in obtaining reliable reports of onsets of hearing loss can make duration of deafness unreliable [24,107]. Demographic characteristics of talkers, such as their gender and age, can influence the intelligibility of their speech [64]. For example, women tend to produce more hyperarticulated speech than men, which can be reflected acoustically by greater vowel dispersion or larger vowel space sizes in women than in men [108,109]. Further, older talkers tend to be less intelligible than younger talkers [110], suggesting an age-related component to intelligibility of talkers (and potentially of listener ratings). Finally, examining individual differences in inner speech among talkers may provide interesting insight into the neural and cognitive mechanisms underlying the production–perception link [111]. Inner speech is inherently related to error monitoring, which entails the integration of auditory feedback into motor planning [112]. Therefore, a more thorough investigation of the relationship between inner speech, phonological processing, and motor planning among CI users may be informative.

Subjective intelligibility ratings may be a promising tool for future clinical and research use, but there are open questions about what constructs these ratings represent. Specifically, individual differences among NH raters may have influenced how they rated speech from the adult CI talkers in the current study. Familiarity with deaf speech has been shown to influence intelligibility, such that listeners who have more experience listening to talkers with HL find their speech more intelligible than listeners with little experience [113]. Cognitive–linguistic factors, like working memory capacity and receptive vocabulary, can also influence intelligibility, especially in adverse listening conditions [25,95,114,115,116]. Further, listeners’ biases, attitudes toward aspects of the talker’s perceived identity, and expectations about how speech from a given talker will sound are associated with intelligibility and other measures of speech perception [117,118,119,120]. These associations reveal a potential limitation of the use of intelligibility ratings, in that they are necessarily subjective and likely depend on a range of listener factors. Therefore, the use of these ratings in future work should be paired with supplemental questionnaires or tasks to quantify some of these properties among NH raters and clinicians to determine their clinical utility.

### 4.5. Clinical Implications

Despite the exploratory, cross-sectional nature of the current study, the results may have clinical relevance for adult CI users. Our finding that subjective intelligibility ratings of CI users’ speech is related to their sentence recognition accuracy expands upon findings from longitudinal work demonstrating the utility of subjective impressions of speech intelligibility both in predicting long-term outcomes [69] and in tracking improvements over the first two years post-activation [57,90]. Although these subjective impressions can be influenced by, for example, listener biases and social attitudes toward a talker or social group [120,121], these subjective metrics may still provide clinicians with useful information that they can use to predict (pre-CI) or explain (post-CI) CI users’ speech recognition outcomes.

Current clinical interventions and auditory rehabilitation strategies for adult CI users primarily target speech recognition, but our findings provide preliminary support that interventions targeting speech production could potentially both enhance speech recognition and influence broader spoken communication outcomes. Regardless of the reasons underlying differences in subjective ratings of intelligibility across talkers or listeners, speech that is evaluated by a listener as being difficult to understand can evoke negative judgments from peers; for example, speech spoken by adolescent CI users is rated more negatively on personality traits related to competence and friendship skills than that of NH peers [122,123]. Though the burden of compensating for these judgments should not necessarily be placed on the CI users themselves, listener biases and judgements may contribute to social isolation experienced by adults with hearing loss, potentially impacting their quality of life [124,125,126]. Therefore, clinicians may be able to track a combination of acoustic and perceptual metrics related to speech production over time to supplement current clinical measures of speech recognition. The feasibility of incorporating measures of speech production intelligibility into clinical assessments should be more thoroughly evaluated, as time constraints and the need for raters may be prohibitive. However, automated acoustic measures may be able to serve as proxies for perceptual judgements, and their development should be further explored.

## 5. Conclusions

The current study examined the relations among speech intelligibility, vowel dispersion, and speech perception outcomes in adult CI users. These exploratory results reveal a potential association between subjective ratings of the intelligibility of CI users’ speech and a measure of sentence recognition accuracy. The marginal but non-significant relationship between intelligibility ratings and phonological processing suggests that phonological processing may be a shared mechanism that contributes to both production and perception performance among adult CI users, but future work with larger sample sizes is necessary to draw conclusions about the nature of this relationship. Similarly, CI users with more dispersed vowel spaces also tended to demonstrate higher speech recognition accuracy than those with less dispersed vowel spaces, but this relationship was not significant. Finally, vowel dispersion was unrelated to phonological processing and did not predict subjective intelligibility ratings. Taken together, these preliminary findings lay the groundwork for future investigation into the use of speech intelligibility as a predictor of speech recognition outcomes and the role of phonological processing in the speech production–perception link among post-lingually deafened adult CI users.

## Figures and Tables

**Table 1:** Demographic characteristics of cochlear implant (CI) users. YR = years, HA = hearing aid, HL = hearing loss, N/A = not applicable. Duration of deafness was defined as the difference in years between patient-reported onset of hearing loss and age at first CI.

Subject	Gender	Age (YR)	Side of Implant	HA in Contra-Lateral Ear	Etiology of HL	Age at First CI (YR)	Duration of Deafness (YR)	Years of CI Use
CI1	Woman	65	Bilateral	N/A	Genetic	54	13	11
CI2	Woman	57	Left	Yes	Genetic	48	41	9
CI3	Woman	69	Bilateral	N/A	Otosclerosis	56	41	13
CI4	Woman	76	Left	No	Autoimmune	68	23	8
CI5	Man	59	Bilateral	N/A	Sudden (idiopathic)	57	2	2
CI6	Woman	62	Right	Yes	Sudden (idiopathic)	56	29	6
CI7	Man	66	Left	No	Meniere’s	60	46	6
CI8	Woman	35	Left	No	Physical trauma	31	18	4
CI9	Woman	64	Left	Yes	Genetic	59	50	5
CI10	Man	56	Bilateral	N/A	Unknown	45	Unknown	11
CI11	Woman	24	Bilateral	N/A	Unknown	19	Unknown	5
CI12	Man	67	Right	Yes	Physical trauma	60	48	7
CI13	Man	73	Right	Yes	Genetic	60	20	13
CI14	Woman	49	Right	Yes	Genetic	39	Unknown	10
CI15	Woman	56	Right	Yes	Unknown	48	Unknown	8

**Table 2:** A count of the total number of words per CI user (talker) that were acoustically analyzed and used as stimulus materials for the intelligibility rating task. Counts are provided separately for each of the four vowels included.

	Vowel				
Talker	æ	ɑ	i	u	Total
CI1	18	23	18	15	74
CI2	18	26	21	16	81
CI3	15	19	20	13	67
CI4	13	22	19	16	70
CI5	15	24	21	16	76
CI6	15	18	17	14	64
CI7	15	22	17	14	68
CI8	17	21	21	13	72
CI9	15	23	17	14	69
CI10	15	23	17	13	68
CI11	16	20	21	16	73
CI12	16	23	22	15	76
CI13	12	22	20	15	69
CI14	15	23	18	12	68
CI15	15	24	20	14	73
Total	230	333	289	216	1068

**Table 3:** A summary of mean intelligibility ratings, vowel dispersion, PRESTO accuracy, and TOWRE-2 scores, provided separately for each CI user. Values in parentheses are standard deviations.

Subject	Intelligibility Rating	Vowel Dispersion (Bark)	PRESTO Accuracy (Percent Correct)	TOWRE-2 Score (Total Words + Nonwords Correctly Reported)
CI1	71.0 (27.6)	3.29 (0.74)	86.6	129
CI2	71.9 (26.5)	2.37 (0.46)	81.4	136
CI3	63.6 (27.8)	2.81 (0.44)	74.1	124
CI4	72.9 (25.4)	2.30 (0.63)	65.0	131
CI5	72.7 (26.3)	2.50 (0.59)	84.4	136
CI6	77.8 (21.7)	1.82 (0.36)	69.4	121
CI7	84.0 (19.2)	2.47 (0.32)	81.2	147
CI8	68.6 (27.1)	2.67 (0.91)	72.8	157
CI9	56.9 (32.6)	2.88 (0.81)	69.9	131
CI10	51.0 (32.0)	2.33 (0.58)	51.6	84
CI11	78.4 (23.5)	2.37 (0.37)	84.7	126
CI12	52.9 (31.5)	1.89 (0.41)	47.6	132
CI13	42.9 (31.2)	2.01 (0.52)	67.7	125
CI14	57.6 (33.2)	2.54 (0.87)	60.2	111
CI15	67.3 (27.5)	1.89 (0.57)	64.9	131
Mean	65.9 (11.5)	2.41 (0.41)	70.8 (11.9)	128.1 (16.2)

**Table 4:** Results from Pearson’s correlations between measures of speech production (intelligibility ratings and vowel dispersion), speech recognition (PRESTO), and phonological processing (TOWRE-2). Bolded comparisons are significant after FDR corrections.

	Sentence Recognition (PRESTO) Accuracy	Phonological Processing (TOWRE-2)
Intelligibility Ratings	**r = 0.67**	r = 0.47
	***p* = 0.024**	*p* = 0.090
Vowel Dispersion	r = 0.50	r = 0.13
	*p* = 0.098	*p* = 0.63

**Table 5:** Model output predicting intelligibility rating from vowel dispersion with covariates for lexical frequency and neighborhood density. *p*-values < 0.05 are denoted with an asterisk.

Effect	Estimate	Error	T-Value	*p*-Value
Intercept	55.56	8.83	6.30	<0.0001 *
Vowel Dispersion	−0.32	0.46	−0.69	0.49
Lexical Frequency	4.60	1.74	2.65	0.009 *
Neighborhood Density	0.001	0.23	0.001	0.99

## Data Availability

The raw data supporting the conclusions of this article will be made available by the authors on request. The data are not publicly available to protect the privacy of the participants.
